# Relationship Between Obesity, Insulin Resistance, and Gastrointestinal Disorders Among Adult Patients With Established Cardiovascular Disease

**DOI:** 10.7759/cureus.115506

**Published:** 2026-08-31

**Authors:** Abdul Moqeet, Sindhu Nanjiani, Muhammad Imran Farooq, Ali Salman, Muhammad Faris Khan, Nida Gull Ashiq, Abdul Wahab

**Affiliations:** 1 Emergency Department, Leighton Hospital, Mid Cheshire Hospitals NHS Foundation Trust, Crewe, GBR; 2 General Practice Department, Liaquat University of Medical and Health Sciences, Jamshoro, PAK; 3 Cardiology Department, Chorley and South Ribble District General Hospital, Chorley, GBR; 4 Internal Medicine Department, Warrington Hospital, Warrington, GBR; 5 Internal Medicine Department, Kazakh National Medical University, Almaty, KAZ; 6 Gastroenterology Department, Sheikh Zayed Hospital, Lahore, Lahore, PAK; 7 Internal Medicine/Family Medicine Department, Faisal Hospital, Faisalabad, PAK

**Keywords:** cardiovascular disease, dietary fiber, fatty liver disease, homa-ir, insulin resistance, nutrition and metabolism, obesity

## Abstract

Background

Obesity and insulin resistance (IR) are major cardiometabolic abnormalities that frequently coexist with cardiovascular disease (CVD) and may contribute to gastrointestinal (GI) morbidity.

Objective

The objective of this study is to determine the relationship between obesity, insulin resistance, and gastrointestinal disorders, including gastroesophageal reflux symptoms, dyspepsia, abdominal bloating, constipation, diarrhea/irritable bowel symptoms, fatty liver disease, and gallstones, among adult patients with established cardiovascular disease.

Methodology

This cross-sectional analytical study was conducted at the Department of Gastroenterology of Sheikh Zayed Hospital, Lahore, from June 2025 to December 2025, among 216 adult patients with established cardiovascular disease. Cardiovascular disease was confirmed from documented medical records and previous cardiology evaluation, including a history of coronary artery disease, myocardial infarction, angina, percutaneous coronary intervention, coronary artery bypass grafting, or ischemic heart failure. Where available, the diagnosis was supported by relevant investigations such as electrocardiography, echocardiography, cardiac biomarkers, and coronary angiography. Demographic data, cardiovascular history, anthropometric measurements, laboratory parameters, and gastrointestinal symptoms were recorded using a structured proforma.

Results

The mean age of patients was 56.8 ± 10.9 years, and 130 (60.2%) were men. Obesity was present in 132 (61.1%) patients, central obesity in 145 (67.1%), and insulin resistance in 142 (65.7%). Overall, gastrointestinal disorders were present in 128 (59.3%) patients. The most common gastrointestinal complaints were gastroesophageal reflux symptoms, 72 (33.3%); dyspepsia, 64 (29.6%); fatty liver, 61 (28.2%); and abdominal bloating, 58 (26.9%). Patients with gastrointestinal disorders had significantly higher body mass index (BMI), waist circumference (WC), fasting blood glucose (FBG), fasting insulin, and homeostatic model assessment of insulin resistance (HOMA-IR) values compared to those without gastrointestinal disorders (p < 0.001).

Conclusion

Gastrointestinal disorders were common among adult patients with established cardiovascular disease and were significantly associated with obesity and insulin resistance.

## Introduction

Cardiovascular disease (CVD) is still one of the leading causes of mortality and morbidity in the world. The World Health Organization (WHO) reports that CVDs are the leading cause of death in the world, contributing to an estimated 19.8 million deaths in 2022, which is approximately a third of all deaths in the world [1]. Many of these are in low- and middle-income countries, where delayed diagnosis and limited preventive care, poor metabolic control, and unequal access to health services contribute to the disease burden [2]. For adults with a diagnosis of CVD, the presence of other metabolic and gastrointestinal (GI) comorbidities can hurt the clinical outcome, lead to more hospital visits, make medication adherence more difficult, and decrease quality of life. Obesity is a large, modifiable risk factor for cardiovascular disease and has long been associated with hypertension, dyslipidemia, type 2 diabetes mellitus, systemic inflammation, endothelial dysfunction, and atherosclerosis [3]. The global obesity problem has rapidly grown during the past decades, and the World Obesity Federation estimates that the number of adults with obesity could increase from 0.81 billion in 2020 to 1.53 billion by 2035. This is especially of concern as obesity is not a single disease but rather a precursor to a complex of abnormalities in metabolism, which have direct effects on cardiovascular, hepatic, pancreatic, and gastrointestinal function [4].

One of the key metabolic abnormalities associated with obesity and cardiovascular disease is insulin resistance (IR). It involves a diminished biological insulin sensitivity of peripheral tissues, which leads to an increase in insulin secretion (hyperinsulinemia), an increase in glucose metabolism (hyperglycemia), and an increase in free fatty acid flux, along with oxidative stress, inflammation, and dysregulated lipid metabolism [5]. There is a close association between metabolic syndrome, which involves central obesity, insulin resistance, hypertension, and dyslipidemia, and atherosclerosis, cardiovascular disease, and type 2 diabetes mellitus. Insulin resistance can be a factor in the progression of established CVD by exacerbating vascular inflammation, endothelial dysfunction, plaque instability, and thrombogenic risk in these patients [6]. Thus, it is clinically relevant to find insulin resistance in cardiac patients, even in those who are not developing overt diabetes. Obesity and insulin resistance are also closely related to the gastrointestinal system. Excess adiposity and visceral obesity are linked to various gastrointestinal diseases such as gastroesophageal reflux disease (GERD), functional dyspepsia, altered bowel habits, gallstone disease, nonalcoholic fatty liver disease (NAFLD), and metabolic dysfunction-associated steatotic liver disease [7]. Findings indicate that abdominal visceral obesity is associated with gastrointestinal conditions such as reflux symptoms and functional dyspepsia, and metabolic dysfunction is associated with liver fat accumulation and chronic low-grade inflammation. While these disorders can present themselves clinically independently of cardiovascular disease, there are common biological pathways shared among these disorders, such as inflammation, autonomic dysfunction, disturbed gut microbiota, impaired bile acid metabolism, oxidative stress, and hormonal changes [8].

Of the gastrointestinal clinical features, fatty liver disease is particularly significant as it is the hepatic expression of metabolic dysfunction. Patients with NAFLD are more likely to be obese, diabetic, and hypertensive and have cardiovascular risk. The 2024 systematic review and meta-analysis from Pakistan found a high prevalence of NAFLD in the context of metabolic diseases such as diabetes, hypertension, and obesity [9]. This is clinically important since fatty liver disease is not only a liver disease but also an indicator of systemic metabolic risk and often linked to adverse cardiovascular events. However, in adult cardiac patients, obesity, insulin resistance, and gastrointestinal disorders may constitute a phenotype that should be evaluated even more comprehensively [10]. There is a likely bidirectional relationship between obesity, insulin resistance, and gastrointestinal disorders. Important mechanisms through which obesity and insulin resistance may contribute to gastrointestinal disease are high intra-abdominal pressure, delayed gastric emptying, motility changes, hepatic fat deposition, gallstone formation, and inflammatory changes [11]. Meanwhile, gastrointestinal disorders can impair cardiometabolic health through decreased physical activity, altered food consumption, drug tolerance, and chronic inflammatory load. For instance, reflux symptoms, dyspepsia, bloating, constipation, fatty liver disease, and abdominal discomfort can impact the ability to take cardiac medications and follow dietary management and exercise guidelines [12]. This interaction is even more clinically relevant in patients with established CVD where long-term risk reduction relies on the strict control of weight, glucose metabolism, lipid profile, blood pressure, and lifestyle factors [13].

Objective

The objective of this study is to determine the relationship between obesity, insulin resistance, and gastrointestinal disorders, including gastroesophageal reflux symptoms, dyspepsia, abdominal bloating, constipation, diarrhea/irritable bowel symptoms, fatty liver disease, and gallstones, among adult patients with established cardiovascular disease.

## Materials and methods

This cross-sectional analytical study was conducted at the Department of Gastroenterology of Sheikh Zayed Hospital, Lahore, from June 2025 to December 2025, among 216 adult patients with established cardiovascular disease. The sample size was calculated using the WHO sample size calculator formula for estimating a population proportion. Obesity was taken as the main reference variable because it was one of the key exposure variables in this study. A previous Pakistani study among patients with coronary heart disease reported an obesity prevalence of 52.5%. This proportion is close to 50%, which provides a conservative estimate and yields nearly the maximum sample size for a single-population proportion. Using a 95% confidence level, an anticipated proportion of 52.5%, and a 7% margin of error, the calculated sample size was 196 patients. After adding 10% to account for incomplete data and nonresponse, the final required sample size was 216 participants.

The following formula was used: n = Z² × P(1−P) / d², where n = required sample size, Z = 1.96 at 95% confidence level, P = the anticipated prevalence of obesity among patients with coronary heart disease (52.5% = 0.525), and d = margin of error (7% = 0.07).

The consequent sampling technique was taken as a non-probability sampling technique. The study included adult patients (18 years old or older) who had a diagnosis of cardiovascular disease; documented coronary artery disease; prior myocardial infarction or angina; a history of percutaneous coronary intervention, coronary artery bypass surgery, or heart failure (ischemic); or other physician-diagnosed cardiovascular disease. Those who provided written informed consent were included. Patients with acute critical illness, pregnancy, known malignancy, chronic liver disease (non-metabolic), chronic kidney disease (stage 4 or 5), a history of major gastrointestinal surgery, chronic inflammatory bowel disease, and current use of systemic steroids and those who were unable to provide consent were excluded. Patients who had incomplete clinical and/or laboratory data were also excluded.

Data collection

Patients were recruited from the outpatient department, inpatient ward, and cardiology/medicine unit after being provided with written informed consent. Demographic data, clinical history, cardiovascular diagnosis, comorbidities, medication history, lifestyle factors, anthropometric parameters, laboratory parameters, and gastrointestinal symptoms were collected via a structured data collection proforma. Cardiovascular disease was considered established when a documented diagnosis was available in the patient's medical record and had been confirmed previously by the treating cardiology or medical team. Confirmation was based on the available clinical history and relevant cardiovascular investigations recorded in the patient file, including electrocardiography, echocardiography, cardiac biomarkers, coronary angiography, CT coronary angiography, or stress testing, where applicable. Patients with documented coronary artery disease, previous myocardial infarction, angina, prior percutaneous coronary intervention, coronary artery bypass grafting, or ischemic heart failure were considered to have established cardiovascular disease.

Demographic factors were age, gender, residence, occupation, smoking status, and physical activity. Cardiovascular-related variables included the type of cardiovascular disease, the duration of disease, a history of myocardial infarction, angioplasty, bypass surgery, hypertension, diabetes mellitus, dyslipidemia, and current cardiac medications. Gastrointestinal disorders were assessed primarily through clinical history, reported symptoms, and a review of available previous diagnoses and investigations. Measurements that were obtained included anthropometric measurements such as height, weight, body mass index (BMI), waist circumference (WC), and waist-hip ratio (WHR). Body mass index (BMI) was computed by dividing weight (kg) by height (m²). Asian BMI cutoff values were used to classify obesity, with a BMI of ≥25 kg/m² defined as obese. Waist circumference was used to determine central obesity. A detailed medical and medication history was obtained from each participant. Medication use included aspirin, other antiplatelet agents, nonsteroidal anti-inflammatory drugs, anticoagulants, proton-pump inhibitors, lipid-lowering agents, antihypertensive drugs, and glucose-lowering medications. Previous medical history included diabetes mellitus, hypertension, dyslipidemia, thyroid disease, and other relevant chronic illnesses. Fasting blood glucose (FBG) and fasting serum insulin were used to evaluate insulin resistance. Homeostatic model assessment of insulin resistance (HOMA-IR) was computed as follows: HOMA-IR = fasting insulin × fasting glucose / 405.

If fasting glucose was measured in mg/dL, then the above formula was used. Insulin resistance (IR) was assessed by a higher HOMA-IR value.

History, clinical symptoms, available previous diagnosis, and appropriate investigations (where applicable) were used to assess gastrointestinal disorders. Gastroesophageal reflux symptoms, dyspepsia, bloating, abdominal pain, constipation, diarrhea, nausea, fatty liver disease, and gallbladder-related symptoms were all gastrointestinal symptoms. Where available, patients were also included if ultrasonographic evidence of fatty liver or gallstones was present. The primary independent variables were obesity, central obesity, and insulin resistance. The dependent variable was the occurrence of gastrointestinal disorders in patients with a definite cardiovascular disease. Other factors evaluated were age, gender, diabetes mellitus, hypertension, dyslipidemia, smoking, physical activity, the duration of cardiovascular disease, and medication intake. A structured proforma was previously designed and used for collecting data for demographics, cardiovascular history, anthropometric measures, laboratory results, insulin resistance assessment, gastrointestinal symptoms, imaging results, and final diagnosis. The participant flow chart is presented in Figure 1.

**Figure 1:**
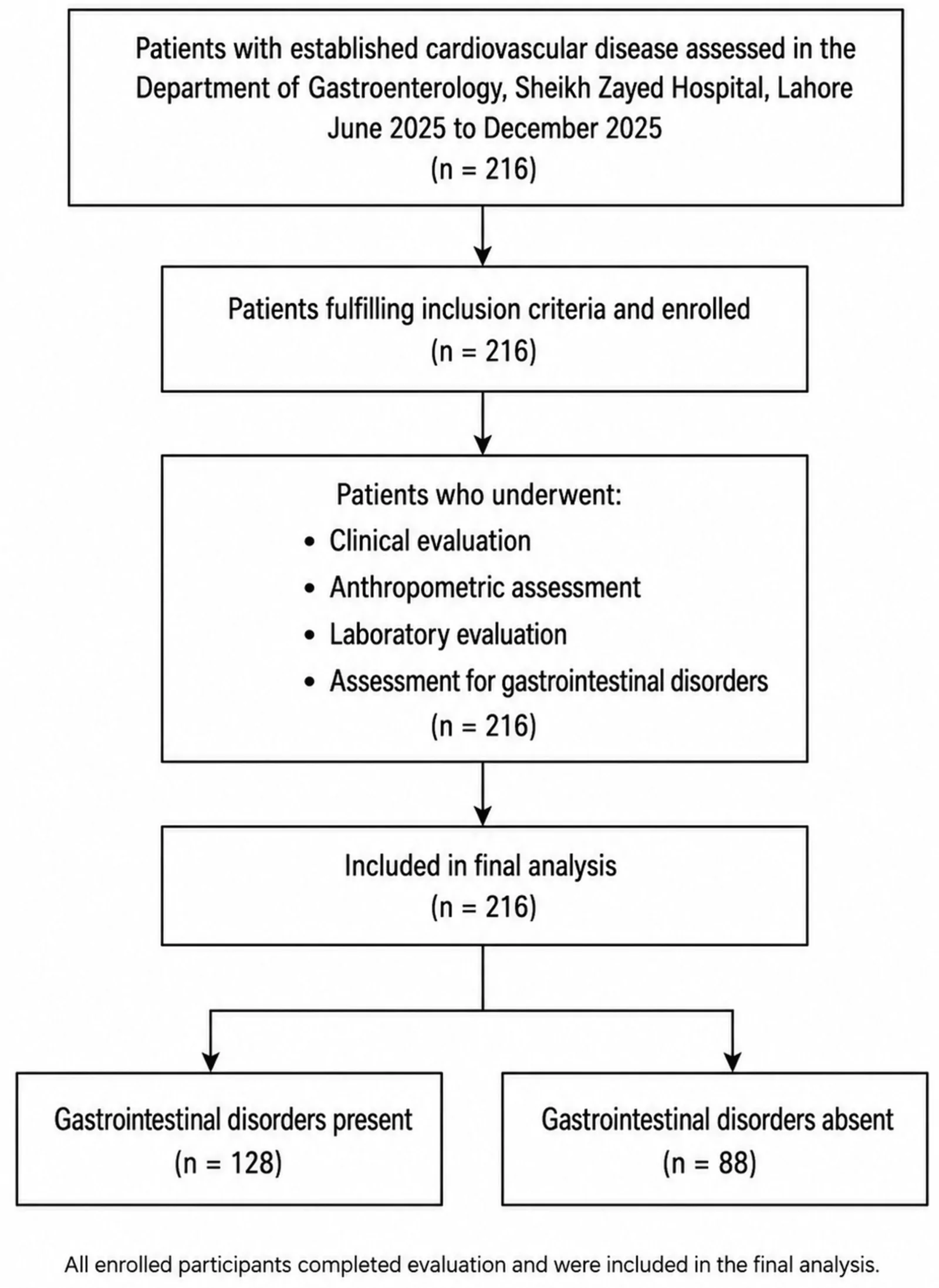
Flow chart of participant recruitment and analysis

Statistical analysis

The data were entered and analyzed using SPSS version 26 (IBM Corp., Armonk, NY). All quantitative variables were presented as mean ± standard deviation, including age, BMI, waist circumference, fasting blood glucose, fasting insulin, HOMA-IR, and the duration of disease. Qualitative variables such as gender, obesity status, central obesity, diabetes mellitus, hypertension, dyslipidemia, smoking, and gastrointestinal disorders were presented as frequencies and percentages. BMI and waist circumference were correlated with gastrointestinal symptoms and disorder burden using correlation analysis. Binary logistic regression was used to investigate whether obesity and insulin resistance were independent predictors of gastrointestinal disorders, controlling for age, gender, diabetes, hypertension, and dyslipidemia. Statistical significance was defined as a p-value of <0.05.

## Results

The mean age of the patients was 56.8 ± 10.9 years. Most participants were men, 130 (60.2%), while 86 (39.8%) were women. The mean body mass index was 28.6 ± 4.9 kg/m², and the mean waist circumference was 96.4 ± 11.8 cm. Obesity was present in 132 (61.1%) patients, while central obesity was observed in 145 (67.1%). Hypertension was the most common comorbidity, present in 151 (69.9%) patients, followed by dyslipidemia in 124 (57.4%) and diabetes mellitus in 109 (50.5%) (Table 1).

**Table 1:** Baseline characteristics of study participants SD, standard deviation; HOMA-IR, homeostatic model assessment of insulin resistance

Variable	Frequency/mean ± SD
Total patients	216
Age, years	56.8 ± 10.9
Male gender	130 (60.2%)
Female gender	86 (39.8%)
Body mass index, kg/m²	28.6 ± 4.9
Waist circumference, cm	96.4 ± 11.8
Obesity	132 (61.1%)
Central obesity	145 (67.1%)
Hypertension	151 (69.9%)
Diabetes mellitus	109 (50.5%)
Dyslipidemia	124 (57.4%)
Coronary artery disease	168 (77.8%)
Previous myocardial infarction	72 (33.3%)
Fasting blood glucose, mg/dL	132.4 ± 38.6
Fasting serum insulin, µIU/mL	16.8 ± 8.7
HOMA-IR	5.3 ± 3.2
Insulin resistance	142 (65.7%)
Gastrointestinal disorder present	128 (59.3%)

Overall, 128 (59.3%) patients had at least one gastrointestinal disorder. Gastroesophageal reflux symptoms were the most common complaint, reported by 72 (33.3%) patients, followed by dyspepsia in 64 (29.6%), fatty liver on ultrasound in 61 (28.2%), and abdominal bloating in 58 (26.9%). Constipation was present in 38 (17.6%) patients, diarrhea or irritable bowel symptoms in 29 (13.4%), and gallstones in 21 (9.7%) (Table 2).

**Table 2:** Frequency of gastrointestinal disorders among study participants

Gastrointestinal disorder/symptom	Frequency, n (%)
Any gastrointestinal disorder	128 (59.3%)
Gastroesophageal reflux symptoms	72 (33.3%)
Dyspepsia	64 (29.6%)
Fatty liver on ultrasound	61 (28.2%)
Abdominal bloating	58 (26.9%)
Constipation	38 (17.6%)
Diarrhea/irritable bowel symptoms	29 (13.4%)
Gallstones	21 (9.7%)

Patients with gastrointestinal disorders had significantly higher BMI compared to those without gastrointestinal disorders: 29.8 ± 4.8 kg/m² versus 26.9 ± 4.5 kg/m² (p < 0.001). Waist circumference was also significantly higher in patients with gastrointestinal disorders: 99.1 ± 11.4 cm versus 92.5 ± 11.2 cm (p < 0.001). Obesity was more frequent among patients with gastrointestinal disorders than those without gastrointestinal disorders: 91 (71.1%) versus 41 (46.6%) (p < 0.001) (Table 3).

**Table 3:** Association of obesity and insulin resistance with gastrointestinal disorders GI, gastrointestinal; BMI, body mass index; HOMA-IR, homeostatic model assessment of insulin resistance

Variable	GI disorder present, n = 128	GI disorder absent, n = 88	Test statistic	P-value
BMI, kg/m²	29.8 ± 4.8	26.9 ± 4.5	t = 4.53	<0.001
Waist circumference, cm	99.1 ± 11.4	92.5 ± 11.2	t = 4.22	<0.001
Obesity	91 (71.1%)	41 (46.6%)	χ² = 13.17	<0.001
Central obesity	95 (74.2%)	50 (56.8%)	χ² = 7.16	0.007
Diabetes mellitus	74 (57.8%)	35 (39.8%)	χ² = 6.79	0.009
Dyslipidemia	82 (64.1%)	42 (47.7%)	χ² = 5.69	0.017
Fasting blood glucose, mg/dL	140.8 ± 40.2	120.3 ± 32.9	t = 4.11	<0.001
Fasting serum insulin, µIU/mL	18.7 ± 9.1	14.1 ± 7.3	t = 4.11	<0.001
HOMA-IR	5.92 ± 3.15	4.41 ± 2.76	t = 3.73	<0.001
Insulin resistance	96 (75.0%)	46 (52.3%)	χ² = 11.96	0.001

Gastrointestinal disorders were present in 17 (40.5%) patients with a BMI of <23 kg/m², 20 (47.6%) overweight patients, 55 (64.0%) patients with obesity class I, and 36 (78.3%) patients with a BMI of ≥30 kg/m². This association was statistically significant (p < 0.001). Similarly, gastrointestinal disorders were more common among patients with insulin resistance compared to those without insulin resistance: 96 (67.6%) versus 32 (43.2%) (p < 0.001). HOMA-IR of >2.5 was used to define insulin resistance (Table 4) [14].

**Table 4:** Gastrointestinal disorders according to BMI and HOMA-IR status HOMA-IR of >2.5 was used to define insulin resistance BMI, body mass index; HOMA-IR, homeostatic model assessment of insulin resistance

Variable	Total, n	Gastrointestinal disorder present, n (%)	Gastrointestinal disorder absent, n (%)	Test statistic	P-value
BMI category					
Normal/underweight, BMI of <23 kg/m²	42	17 (40.5)	25 (59.5)	χ² = 16.16	<0.001
Overweight, BMI of 23-24.9 kg/m²	42	20 (47.6)	22 (52.4)		
Obese I, BMI of 25-29.9 kg/m²	86	55 (64.0)	31 (36.0)		
Obese II, BMI of ≥30 kg/m²	46	36 (78.3)	10 (21.7)		
Insulin resistance status					
No insulin resistance, HOMA-IR of ≤2.5	74	32 (43.2)	42 (56.8)	χ² = 11.96	<0.001
Insulin resistance, HOMA-IR of >2.5	142	96 (67.6)	46 (32.4)		

Obesity was found to be an independent predictor of gastrointestinal disorders, with obese patients having 2.42 times higher odds of gastrointestinal disorders compared to nonobese patients (adjusted odds ratio {AOR}, 2.42; 95% CI, 1.33-4.39; p = 0.004). Insulin resistance was also an independent predictor, with affected patients having 2.18 times higher odds of gastrointestinal disorders (AOR, 2.18; 95% CI, 1.19-3.99; p = 0.011) (Table 5).

**Table 5:** Logistic regression analysis for predictors of gastrointestinal disorders CI: confidence interval

Predictor	Adjusted odds ratio	95% CI	Test statistic	P-value
Obesity	2.42	1.33-4.39	Wald χ² = 8.42	0.004
Central obesity	1.76	0.96-3.21	Wald χ² = 3.37	0.066
Insulin resistance	2.18	1.19-3.99	Wald χ² = 6.38	0.011
Diabetes mellitus	1.55	0.87-2.76	Wald χ² = 2.21	0.138
Dyslipidemia	1.62	0.91-2.87	Wald χ² = 2.71	0.099
Age > 60 years	1.31	0.74-2.34	Wald χ² = 0.85	0.354
Male gender	0.88	0.50-1.56	Wald χ² = 0.19	0.669

## Discussion

The present study evaluated the associations among obesity, insulin resistance, and gastrointestinal disorders in adult patients with cardiovascular disease. The results revealed that gastrointestinal disorders occurred in a high-risk population, with 128 (59.3%) patients affected. Obesity was present in 132 (61.1%) patients, central obesity in 145 (67.1%), and insulin resistance in 142 (65.7%). The results indicate that there is a high overlap of metabolic and gastrointestinal disorders in adult patients with cardiovascular disease and provide support for the idea that cardiovascular disease should not be considered a purely vascular disease but as a part of a cardiometabolic disorder. Among the GI complaints, gastroesophageal reflux symptoms were reported in 72 (33.3%) patients, followed by 64 (29.6%) who had dyspepsia, 61 (28.2%) who had fatty liver on ultrasound, 58 (26.9%) who had abdominal bloating, 38 (17.6%) who had constipation, 29 (13.4%) who had diarrhea/irritable bowel symptoms, and 21 (9.7%) who had gallstones. This pattern suggests that gastrointestinal complaints and hepatobiliary disorders were prevalent in patients who had established cardiovascular disease. Earlier studies have also linked obesity to a variety of gastrointestinal and liver diseases and conditions such as gastroesophageal reflux disease (GERD), gallstones, and fatty liver disease [15].

The BMI, WC, FBG, fasting blood insulin (FBINS), and HOMA-IR were significantly higher in patients with gastrointestinal disorders than in patients without gastrointestinal disorders. Patients with gastrointestinal disorders had a mean BMI of 29.8 ± 4.8 kg/m² versus 26.9 ± 4.5 kg/m² (p < 0.001) in patients without gastrointestinal disorders. On a similar note, patients with gastrointestinal disorders had a higher waist circumference: 99.1 ± 11.4 cm compared to 92.5 ± 11.2 cm (p < 0.001). The present results indicate that both overall obesity and central obesity were significantly associated with gastrointestinal morbidity. This is a plausible etiological mechanism, since visceral adiposity leads to an increase in intra-abdominal pressure, reflux symptoms, the alteration of gastrointestinal motility, the release of inflammatory cytokines, and the accumulation of fat in the liver [14]. The incidence of gastrointestinal disorders was found to gradually rise among the BMI categories. Gastrointestinal disorders were seen in 17 (40.5%) patients with a BMI of <23 kg/m², 20 (47.6%) overweight patients, 55 (64.0%) patients with obesity class I, and 36 (78.3%) patients with a BMI of ≥30 kg/m². This dose-response relationship further reinforces the relationship between excess body weight and gastrointestinal disease burden. It also implies that the higher the BMI, the higher the risk for cardiovascular complications and the more likely the presence of clinically significant gastrointestinal symptoms and metabolic liver disease in the context of cardiovascular disease [15].

Insulin resistance was also significantly associated with gastrointestinal disorders. Insulin resistance was noted in 96 (75.0%) patients having gastrointestinal disorders and 46 (52.3%) patients without gastrointestinal disorders (p = 0.001). The results of this study indicate that insulin resistance is associated with cardiovascular and glycemic abnormalities, as well as gastrointestinal symptom burden [16]. This connection has been supported by previous research, which has identified insulin resistance as an important metabolic pathway in the link between obesity and cardiovascular disease and metabolic complications. Increased insulin resistance is a key association that is significant with fatty liver disease. Of the 61 patients (28.2%) with fatty liver in the current study, 46 (75.4%) had developed it during the study period; 61 patients (28.2%) had fatty liver (46 {75.4%} developed this during the study period). It is believed that fatty liver disease is one of the liver manifestations of metabolic dysfunction and is strongly associated with obesity, insulin resistance, diabetes, dyslipidemias, and cardiovascular risk [17]. Previous studies have demonstrated that there is an increase in insulin resistance in patients with NAFLD, and insulin resistance is believed to play a key role in the pathogenesis and progression of this condition [18]. In addition, a Pakistani systematic review and meta-analysis also found a high prevalence of NAFLD in patients with metabolic syndrome, diabetes, hypertension, and obesity, further highlighting the relevance of the assessment of fatty liver in Pakistani patients with cardiometabolic disease [19,20].

This study's results have implications for the context of existing cardiovascular disease. Patients with CVD already are under the care of long-term treatment for blood pressure, lipid levels, and glycemic status; antiplatelet therapy; and diet and lifestyle modification. Gastrointestinal issues can make care more challenging, as they lead to greater acceptance of medications, more symptoms, less adherence to eating, and worse quality of life. Reflux symptoms, for instance, or dyspepsia can affect compliance with cardiac medications, and fatty liver and insulin resistance can be a sign of greater systemic metabolic dysfunction. As demonstrated by previous studies, metabolic dysfunction-associated fatty liver disease and NAFLD have been increasingly shown to share pathways to cardiovascular risk, including insulin resistance, inflammation, oxidative stress, and dyslipidemia. The findings also underscore the importance of a comprehensive cardiometabolic evaluation in clinical practice in Pakistan. Cardiovascular disease, obesity, diabetes, and fatty liver disease are emerging public health problems in Pakistan and frequently concurrent in the same patient. The prevalence of obesity, central obesity, insulin resistance, and gastrointestinal problems found in this study is consistent with the importance of routinely assessing the metabolic and gastrointestinal function of cardiac patients and not treating them individually.

Limitations

There were a few limitations to this study. First, this was a cross-sectional study, and therefore, it was not possible to determine causal relationships between obesity, insulin resistance, and gastrointestinal disorders. Second, this study was performed in a single institution, and the results may not be applicable in other healthcare settings. Third, gastrointestinal disorders were the subject of a clinical history, symptoms and the available diagnosis, and a few mild and asymptomatic cases might have been overlooked due to insufficient investigations. Fourth, insulin resistance was estimated by HOMA-IR, but this is not the gold standard measurement. Fifth, diet, physical activity, stress levels, and medication adherence could have affected gastrointestinal symptoms and were not easy to control. Finally, no long-term follow-up occurred; therefore, the evolution of gastrointestinal complications and their effect on cardiovascular outcomes could not be examined. The limitations should now explicitly acknowledge that some gastrointestinal conditions may not have undergone uniform confirmatory testing; that the HOMA-IR threshold has not been specifically validated in Pakistani cardiovascular patients; that potential confounders such as specific medication exposure, thyroid disease, and alcohol or opium use may not have been completely characterized if they were not systematically collected; and that the study was not prospectively powered for multivariable modelling.

## Conclusions

Gastrointestinal disorders were common among adults with established cardiovascular disease. Obesity and insulin resistance remained independently associated with gastrointestinal disorders after adjustment for relevant covariates. Central obesity, diabetes mellitus, and dyslipidemia showed associations in univariate analyses but did not remain statistically significant in the adjusted model. These findings demonstrate an association between cardiometabolic abnormalities and gastrointestinal morbidity; however, causal or temporal relationships cannot be established because of the cross-sectional study design.
